# BL-11C Micro-MX: a high-flux microfocus macromolecular-crystallography beamline for micrometre-sized protein crystals at Pohang Light Source II

**DOI:** 10.1107/S1600577521004355

**Published:** 2021-06-01

**Authors:** Do-Heon Gu, Cheolsoo Eo, Seung-A Hwangbo, Sung-Chul Ha, Jin Hong Kim, Hyoyun Kim, Chae-Soon Lee, In Deuk Seo, Young Duck Yun, Woulwoo Lee, Hyeongju Choi, Jangwoo Kim, Jun Lim, Seungyu Rah, Jeong-Sun Kim, Jie-Oh Lee, Yeon-Gil Kim, Suk-Youl Park

**Affiliations:** a Department of Chemistry, Chonnam National University, Gwangju, Republic of Korea; b Pohang Accelerator Laboratory, Pohang University of Science and Technology, 80 Jigokro-127-Beongil, Pohang, Nam-gu, Gyeongbuk 37673, Republic of Korea; c Institute of Membrane Proteins, Pohang University of Science and Technology, Pohang, Gyeongbuk 37673, Republic of Korea

**Keywords:** beamlines, microfocus, macromolecular crystallography, protein structures, serial crystallography

## Abstract

BL-11C, a new protein crystallography beamline, is an in-vacuum undulator-based microfocus beamline used for macromolecular crystallography at the Pohang Accelerator Laboratory and it was made available to users in June 2017. The beamline is energy tunable in the range 5.0–20 keV to support conventional single- and multi-wavelength anomalous-dispersion experiments against a wide range of heavy metals. At the standard working energy of 12.659 keV, the monochromated beam is focused to 4.1 µm (V) × 8.5 µm (H) full width at half-maximum at the sample position and the measured photon flux is 1.3 × 10^12^ photons s^−1^. The experimental station is equipped with a Pilatus3 6M detector, a micro-diffractometer (MD2S) incorporating a multi-axis goniometer, and a robotic sample exchanger (CATS) with a dewar capacity of 90 samples. This beamline is suitable for structural determination of weakly diffracting crystalline substances, such as biomaterials, including protein, nucleic acids and their complexes. In addition, serial crystallography experiments for determining crystal structures at room temperature are possible. Herein, the current beamline characteristics, technical information for users and some recent scientific highlights are described.

## Abstract 2

A new protein crystallography beamline (BL-11C) has been constructed at Pohang Light Source II. The BL-11C beamline allows routine protein-structure determinations under a robot sample-mounting system and room-temperature structure determinations through synchrotron serial crystallography experiments.

## 1. Introduction   

Macromolecular crystallography (MX) beamlines are essential for structural biology investigations because the high-flux X-ray beam makes it easier to determine the structure of protein crystal samples including membrane proteins and perform a high throughput analysis for drug development (Bragg & Bragg, 1913 ▸; Bennett, 2010 ▸). The Pohang Light Source II (PLS-II) has operated two MX beamlines (BL5C and BL7A) (Park *et al.*, 2017 ▸) because its storage ring was upgraded in 2012 (Shin *et al.*, 2013 ▸). PLS-II currently operates at an energy of 3.0 GeV with stored currents up to 400 mA in a top-up mode (Hwang *et al.*, 2014 ▸). To date, 1499 protein structures have been deposited in the Protein Data Bank by using data from the 5C and 7A beamlines. In 2016, over 92 research groups performed investigations using the two beamlines, including users from academic institutions and pharmaceutical companies. However, it was not possible to ensure conformance with the required beam times for all the groups because the number of users was large. Thus, to satisfy the user beam time requirements, we designed and constructed a new micro-MX beamline (BL-11C), which was made available to general users in June 2017.

Based on the strong demand from the Korean MX community, BL-11C was designed for micro-crystallography with a default full beam size of less than 10 µm. The main optics of the beamline contain a double-crystal monochromator (DCM) and a Kirkpatrick–Baez (KB) type focusing mirror, as well as a Pilatus3 6M detector for shutterless data collection. To increase the efficiency of data collection, an automatic mounting and remote system was adopted. Furthermore, to use the 11C micro-focused beam at its full potential, synchrotron serial crystallography (SSX) was introduced for the observations on protein dynamics and physiological structure determination with a minimized radiation damage (Wierman *et al.*, 2019 ▸; de la Mora *et al.*, 2020 ▸).

Thus, this beamline is optimized for structural determination by traditional crystallography and serial crystallography. This article describes the performances and functionalities of the BL-11C beamline to overseas users as well as Korean users.

## 2. Beamline overview   

**Figure 1:**
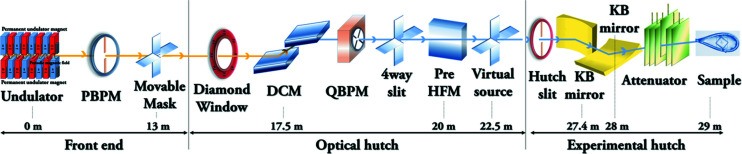
A schematic layout of the 11C Micro-MX beamline. The components are an in-vacuum undulator insertion-device source (ID), a front-end moveable slit (FE slit), a DCM, the first HFM, a horizontal slit as a virtual source, and the second focusing vertical and horizontal mirrors as KB mirrors. The total distance is 29 m from the source to the sample point.

The 11C beamline is illuminated by a hybrid and asymmetric type in-vacuum 1.4 m-long undulator with a periodicity of 20 mm and a minimum operational gap of 12 mm (Table 1 ▸). There are three segments: a front-end for the undulator source; an optical hutch for the DCM, pre-horizontal focusing mirror (pre-HFM) and JJ slit; and an experimental hutch for the KB mirrors, goniometer, detector and sample changer. The optical layout of the 11C beamline is shown schematically in Fig. 1 ▸.

### 2.1. Beamline layout and optics   

The white beam from the undulator source passes through a fixed mask, a one-dimensional photon-beam position monitor (PBPM), a movable mask which reduces the heat load of the downstream optical components and a 200 µm-thick chemical vapor deposition water-cooled diamond window. The diamond window separates a vacuum status between the front-end and the optical hutch, which is visualized by a Basler acA1300-30gm GigE monochrome camera (Basler AG, Ahrensburg, Germany) via a web browser. The maximum brilliance of the undulator source from the camera image was understood to be a coherent core of the radiation. The polychromatic beam is monochromated by an Si(111) DCM, which is cryogenically cooled by a liquid-nitro­gen cryo-cooler system (Oxford Instruments, UK). The monochromator was designed for automated energy changes, which are available for single- or multi-wavelength anomalous-diffraction experiments from 5.0–20 keV by using various metals as well as seleno-me­thio­nine.

The monochromator-derived mono-beam is focused in the horizontal direction by two figured elliptical mirrors, whereas in the vertical direction, the beam is focused by one figured elliptical mirror. The KB mirrors are coated with rhodium and platinum on the mirror surface in halves. Since all the mirrors on this beamline are pre-figured and do not have benders, the beam size is fixed.

**Figure 2:**
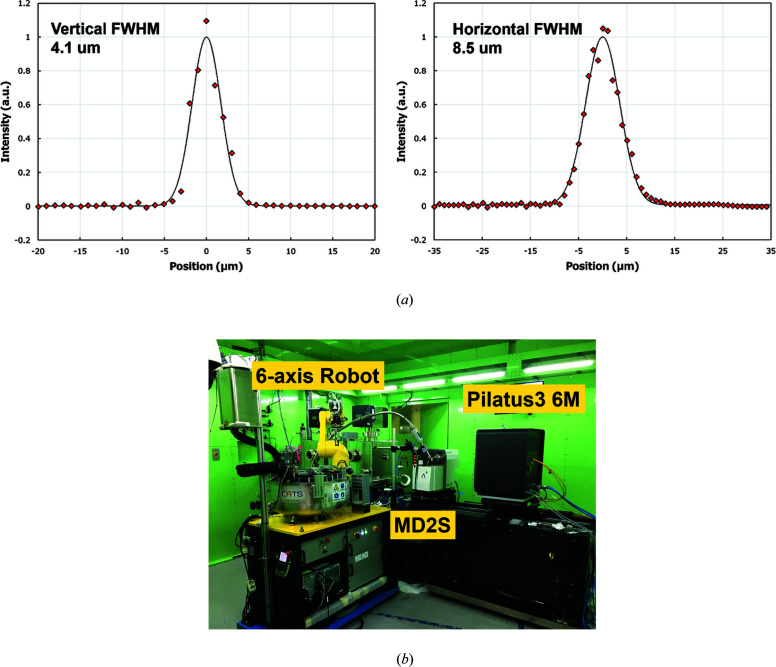
(*a*) Measured vertical and horizontal profiles of the micro-focused beam obtained using a knife-edge-scan setup giving a focus beam 4.1 µm (V) × 8.5 µm (H) in size. The red rhombuses are the measured data whereas the solid lines are the fitted Gaussian profiles. The data were collected with ∼10^12^ photons s^−1^ at a photon energy of 12.659 keV. (*b*) The 11C Micro-MX experimental hutch showing a six-axis robot arm, an MD2S diffractometer and a Pilatus3 6M detector.

The mono-beam is first focused at a JJ slit (JJ X-Ray, Denmark) by the first horizontal mirror (pre-HFM) located in the optics hutch. Then, the horizontally focused beam is diverged as a virtual source for the second focusing KB mirror in an endstation. Ray-tracing using *SHADOW* (Sanchez del Rio & Dejus, 2004 ▸) calculated that the minimum beam size at the sample position is 1.75 µm (V) × 11 µm (H) with 1 × 10^12^ photons s^−1^ flux based on 12.6 keV and 350 mA storage-ring current. After completion of the beamline construction, the measured full beam size was 4.1 µm (V) × 8.5 µm (H) with 1.3 × 10^12^ photons s^−1^ flux at the same energy and current as above [Fig. 2 ▸(*a*)]. The beam size is different from the calculated value but it is usable for collecting data from very small crystals up to 5 µm in the smallest dimension. As the micro-beam is sensitive to external factors of the photon transfer line, considerable attention has been given to constructing a rigid support, removing vibration sources and thermal effects, and monitoring the beam position. Thus, the main optical and experimental components like the DCM, mirrors, the goniometer and the detector are installed above granite bases which are grouted by the micropile method. The experimental hutch, including the KB mirrors, is air conditioned to within 0.1°C using a high-performance air conditioner (Orion precision air processor, Japan), and insulators between the hutch steel plates are adapted as a thermal buffer space. For beam diagnosis, three mono-beam viewers with YAG materials were each installed downstream of the DCM, the pre-HFM and the JJ slit in the optical hutch. In addition, a quadrant beam position monitor (QBPM) (FMB Oxford, UK) was installed to measure beam intensity and position.

### 2.2. Experimental setups   

The endstation is equipped with an MD2S diffractometer (ARINAX, France) with an on-axis crystal-visualization system, a Pilatus 6M detector (DECTRIS, Swiss), an Si-PIN photodiode detector XR-100CR (Amptek, USA) for anomalous-diffraction experiments using X-ray absorption near-edge structure (XANES) analysis and fluorescence measurements, and a sample cryo-cooler Cryojet5 (Oxford Instrument, UK) [Fig. 2 ▸(*b*)]. The MD2S diffractometer also supports the chi-axis movement up to 48° by the mini-Kappa goniometer head (ARINAX, France) (Brockhauser *et al.*, 2013 ▸). The beamline incorporates a robotic sample-transfer system, the cryogenic automated transfer system (CATS) (IRELEC, France), that enables the efficient handling of frozen protein crystals and a cryogenic storage dewar (90 samples) under liquid nitro­gen with an automated re-fill system for the diffraction-quality screening. Thus, samples can be mounted manually or via the CATS six-axis robot arm (IRELEC, France), a Stäubli TX60L six-axis industrial robot (Stäubli Faverges SCA, Faverges, France), using a wet method to prevent icing on the sample. The robotic arm is equipped with an IRELEC flipping gripper (Jacquamet *et al.*, 2009 ▸) for samples stored in SC3 format pucks (Cipriani *et al.*, 2006 ▸). The sample is exchanged in 45 s.

The Cryojet5 maintains the temperature of the sample from 100 K to room temperature. A Colibri X-ray fast shutter (Arinax, France) is connected to the MD2S diffractometer to allow synchronization with its goniometer axis. Downstream of the mirrors is a high-vacuum attenuator box containing a total of eight aluminium foils for 256 combinations with thicknesses of 0.0125, 0.025, 0.05, 0.1, 0.2, 0.4, 0.8 and 1.6 mm. For insertion into the X-ray beam, each foil is mounted on an individual pneumatic actuator, and the composition of the foils in the attenuator box is selected to best match the attenuation required across the energy range 5–18 keV. The MD2S diffractometer is currently equipped with an independently movable penta-aperture (with diameters of 300, 100, 75, 50, 30 and 20 µm), a beam-cleaning capillary and a beam stop. Generally, the 20 µm aperture is used to remove parasitic scattering. Crystals generated from a screening plate are also available for diffraction tests using a crystal-plate holder (Arinax, France). The Pilatus3 6M detector (DECTRIS, Switzerland) is mounted at sample-to-detector distances between 150 mm and 900 mm to cover data collection at 12.659 keV with a resolution between 1.0 Å and 4.1 Å.

### 2.3. Serial crystallography setups   

Typically, injector-based serial crystallography (IBSX) and fixed-target serial crystallography (FTSX) methods have been used for SSX experiments (Panneels *et al.*, 2015 ▸; Weinert *et al.*, 2017 ▸; Wierman *et al.*, 2019 ▸). Both methods are available in the 11C beamline. To perform serial crystallography experiments using the FTSX method, 60 µm-pore-sized nylon mesh (Vision Lab Science, Republic of Korea), polyimide film (Covalue Youngjin Corporation, Republic of Korea), and polyvinyl chloride (PVC) frames were assembled for sample preparation based on a nylon mesh-based sample holder. A detailed description of this FTSX holder assembly is provided by Lee *et al.* (2019 ▸) and Park *et al.* (2020 ▸). During FTSX data collection, raster scanning was performed at 50 µm intervals (Park *et al.*, 2020 ▸). For IBSX experiments, viscous sample-delivery media (*e.g.* lipidic cubic phase, polyacryl­amide and shortening), various syringes including disposable and Hamilton syringes (81065-1710RNR), and a Fusion Touch 100 syringe pump (CHEMYX) are prepared (Park & Nam, 2019 ▸). The details of IBSX experiments were provided by Park & Nam (2019 ▸).

### 2.4. Beamline control and software   

To control beamline motors and signal readout, a system consisting of an integrated graphical interface based on experimental physics and industrial control system (EPICS) input–output controllers (IOCs) was developed for the 11C beamline (Dalesio *et al.*, 1994 ▸).

Crystal mounting and data collection from the MD2S goniometer and Pilatus 6M detector is controlled by *MX Data Collector* (*MxDC*), which was provided by the Canadian Light Source (Fodje *et al.*, 2012 ▸). The modified *MxDC* for the 11C allows centering of crystals in the micro-focused X-ray beam and provides access to the diffraction, the fluorescence-scan experiments, and the beam-alignment setups. Remote access is also available by using the *NoMachine* NX remote desktop-virtualization software system (http://www.nomachine.com/) for experiments and data processing. *HKL2000* (Otwinowski & Minor, 1997 ▸) is generally used for diffraction-data display, refinement and scale. The *Cheetah* (Barty *et al.*, 2014 ▸) and *CrystFEL* (White *et al.*, 2012 ▸, 2016 ▸) programs for X-ray free-electron laser experiments are also available for SSX. Additionally, *SHARP/autoSHARP* (Vonrhein *et al.*, 2007 ▸), *CCP4i* (Winn *et al.*, 2011 ▸) and *Phenix* (Adams *et al.*, 2010 ▸) are installed for molecular replacement and phasing analysis. All the above programs for diffraction analysis are supported by Dell PowerEdge R940 with a Dell EMC X4012 10Gb-T network module and a data-storage capacity of 120 TB

### 2.5. Ancillary facilities   

The 11C beamline can use two high-resolution stereo microscopes with 240× magnification from Leica (US), enabling users to view, analyze, and report their crystals in two and three dimensions. One microscope is in the experimental hutch for rapid manual mounting while the other microscope is located outside of the hutch to fill robot mounting pucks or for variable uses. In addition, there are two incubators to keep the crystals safely at the desired temperature for general (16–22°C) and low (4–12°C) temperatures. The beamline is also supported by a structural biology laboratory that is certified with biosafety level 1. The laboratory is equipped with microscopes, incubators, a refrigerator, clean benches, pH meters, centrifuges, balances and other common laboratory equipment.

## 3. Facility access   

Users are required to submit beam time proposals to access the 11C Micro-MX beamline (three terms per year; submissions are peer-reviewed and scored by the committee) via a website (http://pal.postech.ac.kr) before being granted permission by Pohang Accelerator Laboratory (PAL), as PLS-II is operated by PAL. Beam proposals are either (i) general proposals open to public users or (ii) rapid access (RA) proposals that can be submitted at any time and are intended for important or urgent samples. In particular, the RA beam time is assigned in four weeks as a fast track. In general, 70% of the beam time is distributed among the public users, and the remainder is used for maintenance and upgrades by beamline staff. Since the 11C beamline started operating, the oversubscription problem for MX beam time at PLS-II has been resolved by ratio from 2.0:1 to 1.2:1.

## 4. Highlights   

One of the recent highlights in the 11C beamline was the determination of the Anti-CRISPR (Acr) protein AcrIIC3 using a routine X-ray crystallography method. AcrIIC3 directly inhibits target DNA cleavage of type II-C Cas9 of *Neisseria meningitidis*. The AcrIIC3 protein interacts with the HNH nuclease domain of *N. meningitidis* Cas9 to inhibit its nuclease activity in an allosteric manner (Kim *et al.*, 2019 ▸). Another highlight was the observation of an arginase (zmARG) structure from *Zymomonas mobilis* ZM4 by selenium single-wavelength anomalous-diffraction *de novo* phasing using the helical-scan function to reduce radiation damage (Hwangbo *et al.*, 2019 ▸). The revealed high-resolution crystal structure of zmARG showed that it contains two metal ions in the active site. The metal ions were identified as zinc ions by the XANES and a fluorescence scan. Additionally, regarding Legionnaires’ disease, the Dot/Icm type IVB coupling protein (T4CP) complex was elucidated to examine how the Dot/Icm T4CP complex can recognize a notable number of effector proteins (Kim *et al.*, 2020 ▸). That structural study examined the interaction between the Dot/Icm T4CP subcomplex and substrates.

Moreover, SSX experiments were performed to determine protein structures at room temperature to ultimately capture protein dynamics, which represents a significant challenge in the field. We performed the SSX experiments with ∼20 µm-sized lysozyme crystals at room temperature using IBSX and FTSX methods (Park & Nam, 2019 ▸; Park *et al.*, 2020 ▸). Through both these methods, ∼19600 and 40000 diffraction images were collected during observations of 40 min and 80 min, using the 10 Hz synchrotron X-ray radiation of the 11C beamline. The crystal structures of lysozyme were determined at 1.89 Å and 1.80 Å resolutions, respectively.

## 5. Discussion and conclusions   

The 11C Micro-MX beamline at PLS-II was designed and constructed in 2016 for protein crystallography experiments, and has been operated by domestic and international users in several configurations. It uses a tunable high-flux X-ray source to obtain remarkably strong intensity diffraction results compared with bending-magnet X-ray sources as the undulator provides high beam quality and significantly higher brilliance (Geloni *et al.*, 2015 ▸). The Pilatus 6M detector based on shutterless data collection collects a large amount of data compared with classical charge-coupled devices. In addition, the robotic sample-mounting system is useful for high-throughput experiments. The 11C Micro-MX software has a Linux-based graphical user interface, and various ancillary facilities in the beamline help support high-quality experiments related to raster scan and helical scan, as well as SSX.

X-ray crystallography has been an effective method for revealing the structure of molecules. However, the classical X-ray crystallography experiment to investigate protein structures shows its fundamentally limited capacity for understanding the mechanism of protein dynamics under cryogenic condition. Serial crystallography allows the observations of protein dynamics and physiological structure determinations with minimized radiation damage. Unlike the classical crystallographic method, data collections of SSX are performed with thousands of micro-crystals at room temperature using both the IBSX and FTSX technical methods.

Herein, the described BL-11C beamline features involving the SSX methods will be useful for crystallographers to determine operative dynamic structural information at room temperature ultimately for time-resolved analysis, as well as single-crystal structure determination under cryogenic condition. For a further beamline upgrade, microfluidic chips are being developed to study enzyme dynamics with substrates.

## Figures and Tables

**Table 1:** Beamline details

Beamline name	11C Micro-MX
Source type	In-vacuum undulator 20 (1.4 m short, 20 mm period)
Monochromator	Si(111) DCM
Energy range	5.0–20 keV, user controlled
Mirror	Elliptical KB mirror
Beam divergence at sample position (mrad)	1 (V), 1.5 (H)
Beam flux	1.3 × 10^12^ photons s^−1^
Beam size (FWHM)	4.1 µm (V) × 8.5 µm (H)†
Energy resolution (Δ*E*/*E*)	2 × 10^−4^
Detector	Pilatus3 6M from Dectris
Goniometer	MD2S diffractometer from Arinax

† Theoretical specification for minimum beam size = 1.75 µm (V) × 11 µm (H).
